# Factors Affecting Outcome in Acute Hypertriglyceridemic Pancreatitis Treated with Plasma Exchange: An Observational Cohort Study

**DOI:** 10.1371/journal.pone.0102748

**Published:** 2014-07-21

**Authors:** Jakob Gubensek, Jadranka Buturovic-Ponikvar, Karmen Romozi, Rafael Ponikvar

**Affiliations:** Department of Nephrology, University Medical Center Ljubljana, Ljubljana, Slovenia; University of Szeged, Hungary

## Abstract

**Objectives:**

The optimal therapy for hypertriglyceridemic acute pancreatitis, especially the role of plasma exchange (PE), is not entirely clear. The aim of our large, single-center, observational, cohort study was to analyze the factors affecting outcome in hypertriglyceridemic pancreatitis treated with PE.

**Methods:**

We included 111 episodes of hypertriglyceridemic pancreatitis treated with PE, which occurred in 103 different patients. The Acute Physiology and Chronic Health Evaluation (APACHE) II score, triglycerides, delay to first PE, and PE treatment details were retrospectively obtained from the patients’ records. The main outcome measures were length of hospitalization and in-hospital mortality.

**Results:**

The patients were 47±9 years old and the median APACHE II score at first PE was 4 (inter-quartile range (IQR) 2–7). There was a seasonal variation in the incidence of hypertriglyceridemic pancreatitis, and the recurrence rate was 1.6% per year. Triglycerides at presentation did not correlate with APACHE II or influence the outcome. The mean reduction in triglycerides during PE was 59% (from 44±31 to 18±15 mmol/l), which was twice the reduction observed during conservative treatment (27% daily). The median hospital stay was 16 days (IQR 10–24) and in-hospital mortality was 5%. The median delay to first PE was 35 hours (IQR 24–52), and there was no difference in mortality in the early and late PE groups (7% vs. 6%, p = 0.79). The group with citrate anticoagulation during PE had a significantly lower mortality than the group with heparin anticoagulation (1% vs. 11%, p = 0.04), and citrate was an independent predictor also in the multivariate model (p = 0.049).

**Conclusions:**

PE effectively reduced serum triglycerides faster than could be expected with conservative treatment. The delay in PE therapy did not influence survival. We found that citrate anticoagulation during PE was associated with reduced mortality, which should be confirmed in a randomized study.

## Introduction

Hypertriglyceridemic pancreatitis is an acute pancreatitis that occurs in the presence of severe hypertriglyceridemia and accounts for about 1–7% of all cases of acute pancreatitis in large series [1]–[3]. While mild hypertriglyceridemia is a common epiphenomenon in acute pancreatitis of any etiology [4], [5], severe hypertriglyceridemia above approximately 10–20 mmol/l, the level at which chylomicrons begin to form, is a well-recognized cause of acute pancreatitis [6], [7]. The pathophysiology of hypertriglyceridemic pancreatitis is not well established. It is hypothesized that pancreatic lipase hydrolyzes excess triglycerides into free fatty acids, which are toxic to acinary cells and the vascular endothelium [8]. Additionally, chylomicrons increase the viscosity of blood and impair blood flow in the pancreas, causing ischemia within the pancreas. The proposed mechanisms are supported by animal models showing that hypertriglyceridemia intensifies the course of pancreatitis [9], [10], although in humans the data on the effect of hypertriglyceridemia on the course of pancreatitis are conflicting; some report no difference in the clinical course as compared to pancreatitis from other etiologies [2], while others do report more severe disease [11], [12] and also higher mortality with severe hypertriglyceridemia [13].

The optimal therapy of acute hypertriglyceridemic pancreatitis is not entirely clear. In addition to eliminating oral intake, hydration and analgesia, which constitute the standard therapy of pancreatitis of any etiology, specific therapies have been used to reduce triglyceride levels, including heparin and insulin [8], [14], as well as plasma exchange [15]–[19]. Heparin stimulates the release of endothelial lipoprotein lipase into circulation, while insulin activates lipoprotein lipase, therefore increasing the clearance of chylomicrons from plasma. Plasma exchange (PE), on the other hand, rapidly removes triglycerides from plasma and thus seems to be a very reasonable and effective therapy, although it is not accepted and applied universally, probably due to the absence of randomized studies showing its superiority over conservative management. In its latest guidelines [20], the American Society for Apheresis considers acute hypertriglyceridemic pancreatitis as a category III indication, meaning that the optimal role of PE has not been established and an individualized decision is proposed. Accordingly, a treatment algorithm from a recent review [8] suggests that PE treatment be considered if it is available and the patient can tolerate it. For centers that do use PE, it has been suggested that an early start of treatment might be important [18].

In the long-term management of hypertriglyceridemia and prevention of recurrent pancreatitis attacks, lifestyle changes and dietary modifications play a key role, including abstinence from alcohol, weight reduction and control of diabetes [21]. To further reduce triglyceride levels fibrates, nicotinic acid and omega-3 fatty acids can be used and regular plasma exchange treatment has also been described in the prevention of recurrent attacks [21].

The aim of our large, single-center, observational, cohort study of patients with acute hypertriglyceridemic pancreatitis, treated with PE, was to report on the effectiveness of PE in reducing triglycerides, patients’ outcome and the factors affecting it.

## Methods

### Ethics statement

All data were retrieved retrospectively from medical records and were analyzed in anonymized form. Due to the retrospective and observational nature of the study, specific patients’ consent for the use of anonymized medical data was not sought. The study was approved by the National Medical Ethics Committee (Ref. no. 83/08/13).

### Study population

We included in this retrospective observational study all episodes of acute hypertriglyceridemic pancreatitis referred to the University Medical Center Ljubljana for PE treatment between Jan. 1^st^, 2003 and June 30^th^, 2013. Patients were referred for PE when severe hypertriglyceridemia above 10–20 mmol/l was present in the setting of acute pancreatitis.

### Conservative treatment and plasma exchange procedures

In addition to standard therapy with fasting, infusions and analgesics, PE treatment was instituted as soon as possible (PE treatment is available at our Center around the clock). Low molecular weight heparins were only used for thrombosis prophylaxis at the discretion of the treating physician, and insulin was only used when overt hyperglycemia was present. As a rule, PE was performed daily until triglyceride levels were below 10 mmol/l. During each PE, one (rarely up to two) estimated plasma volume was exchanged and replaced with a bicarbonate-based electrolyte solution with 30 g/l albumin added. Vascular access was obtained with a double- or single-lumen catheter usually placed in the femoral vein; peripheral veins were used for the return of blood in some cases. Anticoagulation during PE was achieved with unfractionated heparin or trisodium citrate (either 4% or 15% solution; the protocol included intravenous calcium substitution).

### Data collection and statistical methods

The patients’ charts were retrospectively examined to obtain data on the approximate delay between the onset of pain and presentation to the emergency department, the delay to first PE, and the Acute Physiology and Chronic Health Evaluation II (APACHE II) score at first PE. Length of hospital stay and patient survival until hospital discharge were recorded as the main outcome measures. The reduction in triglycerides during PE sessions was calculated using values immediately after PE (sometimes available) or on the next day. In some cases, usually after several PEs, patients still had triglycerides above 10 mmol/l, but did not receive PE for more than 24 hours and had another triglyceride measurement done. We collected all such 24-hour episodes and calculated the percentage of daily reduction in triglycerides with conservative management and compared it to the reductions during PE. All PE procedures were reviewed and the PE parameters were recorded, as well as any eventual complications during PE treatment. Hypotension was defined as blood pressure dropping to 90/60 mmHg and significant hypocalcemia as ionized calcium dropping to ≤0.90 mmol/l during the procedure.

Since all cases from a single-center were included and the observation period was long, we estimated the recurrence rate for hypertriglyceridemic pancreatitis needing PE treatment by dividing the number of recurrent cases with the total observation period for all patients. We checked all available hospital records for patients’ death from other causes during the study period, but we did not actively check the patients’ vital status at the end of the observation period and assumed the patients were alive; this may have decreased the reported recurrence rate.

To evaluate the potential factors affecting patient outcome, the episodes of pancreatitis in which patients survived and those in which patients died were compared. Mortality was compared in the high and low triglycerides groups as well as in the early and late PE groups, which were formed by splitting the whole group at the median values of respective variables. Mortality was also compared in the severe (i.e. APACHE II ≥8 [22]) and mild pancreatitis groups, as well as in the groups with citrate and heparin anticoagulation during PE. Only two patients were treated with both heparin and citrate anticoagulation, and were excluded from this analysis; one of them died.

Data is presented as mean ± standard deviation, median and inter-quartile range (IQR) or percentage, as appropriate. Statistica 7.0 (StatSoft, Inc., Tulsa, USA) was used for statistical analysis. Continuous variables were compared between groups using the Student’s T test and dichotomous variables using the Chi-square test, or the Fisher exact test (two-tailed) in cases of small frequencies. A Kaplan-Meier survival analysis was used to estimate the percentage of patients without recurrence.

## Results

### Patients’ characteristics

**Figure 1:**
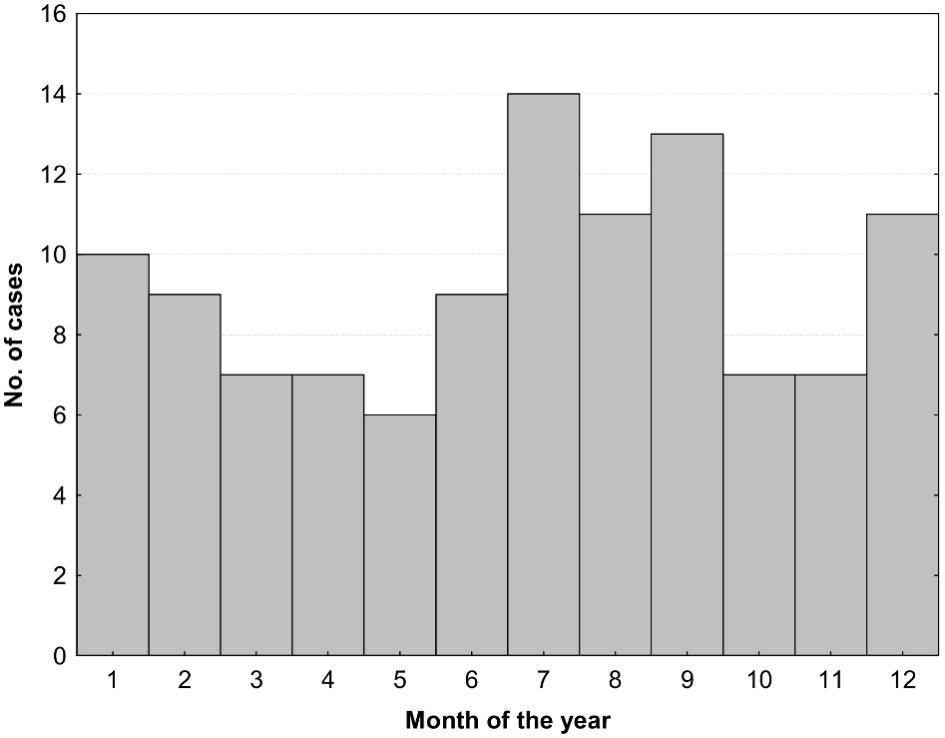
Seasonal variation in the incidence of acute hypertriglyceridemic pancreatitis.

There were 111 episodes of acute hypertriglyceridemic pancreatitis occurring in 103 different patients. The main patients’ characteristics are given in Table 1, and the details are available in the Table S1. In 35 cases (32%), patients had had a previous episode of pancreatitis, and in 7/35 (20%) cases it was attributed to hypertriglyceridemia and treated with plasma exchange. We have observed a seasonal variation in the incidence of pancreatitis (see Figure 1) with two peaks, one around the New Year and the other in summer. During a median 3.2 years of observation, there were 7 cases (out of 111) of recurrent hypertriglyceridemic pancreatitis in 4 different patients (1–3 recurrences per patient), resulting in a 1.6% per year recurrence rate. When estimated with a Kaplan-Meier analysis, 96% of patients at one year and 93% at 5 years were recurrence-free. In 94 patients, the time delay to first PE could be established; the median delay was 35 hours, of which the time from the onset of pain until presentation to the emergency department was 17.5 hours (see Table 1). Triglycerides were documented as having fallen below 10 mmol/l within 2 days (IQR 1–3) of treatment with PE; data was only available in 76 cases, while in other cases the last documented triglycerides were slightly above 10 mmol/l and were not included in this calculation.

**Table 1:** Main patients’ characteristics and outcome.

Parameter	Value
N	111
Age (years)	47±9 (range 19–73)
Male gender	97 (87%)
Diabetes mellitus:	34 (31%)
• already known	21 (19%)
• newly diagnosed	13 (12%)
Alcohol abuse	24 (22%)
Known hyperlipidemia	51 (46%)
Triglycerides (mmol/l)	56±34 (range 8–183)
APACHE II at first plasma exchange	4 (IQR 2–7)
Delay from start of pain to hospital (hours)	17.5 (IQR 10.5–24)*
Delay from start of pain to first PE (hours)	35 (IQR 24–52)*
Hospital stay (days)	16 (IQR 10–24)
In-hospital mortality	5%

*N = 94.

### Plasma exchange treatment

Altogether 191 PEs were performed. In the majority of cases (56%), only one PE was performed; the median number of PEs per patient was 1 (IQR 1–2) and the maximum was 5. The mean volume of plasma exchanged per procedure was 4453±1277 ml over 3.7±1.6 hours. Citrate was used as an anticoagulant in the majority (73%) of procedures, and unfractionated heparin in the rest. Regarding complications, in 7 procedures (3.7%) the plasma filter was replaced due to increased transmembrane pressure, and in 2 procedures (1%) the filter clotted and the procedure was restarted. There were 5 (2.6%) cases of hypotension and one case of severe gastrointestinal bleeding after PE with heparin anticoagulation. During 139 PEs with citrate anticoagulation, there were 5 episodes (3.6%) of significant hypocalcemia during PE. The full data set is available in the Table S2.

### Reduction of triglycerides: conservative treatment vs. plasma exchange

**Figure 2:**
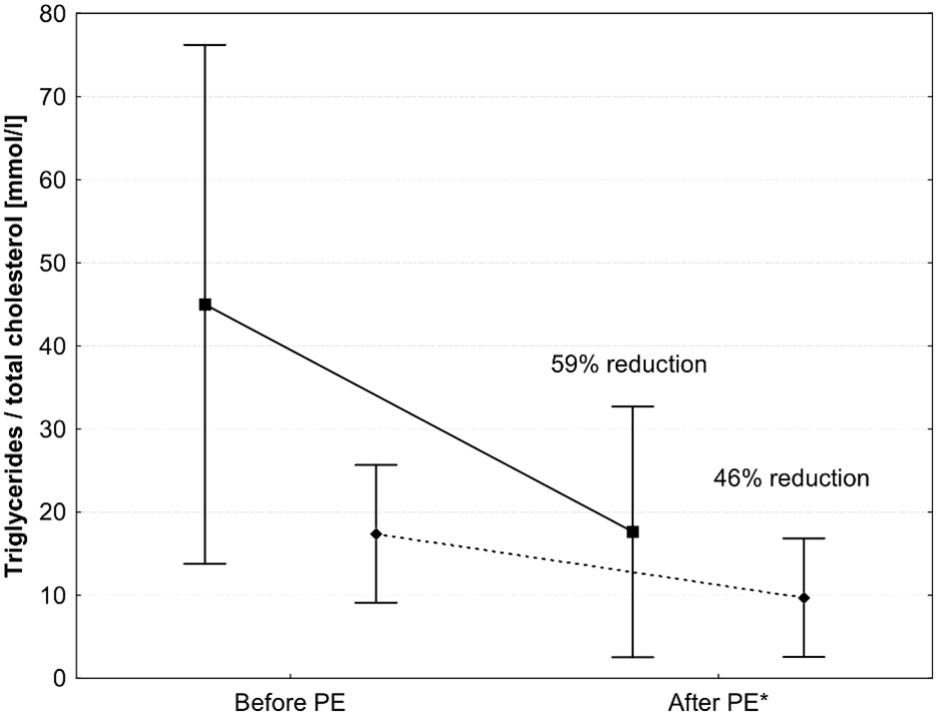
Reduction of triglycerides (squares and full line) and cholesterol (circles and dotted line) during plasma exchange (PE) treatments. Data is shown as mean ± standard deviation. *After PE = immediately after PE or on the next day.

**Figure 3:**
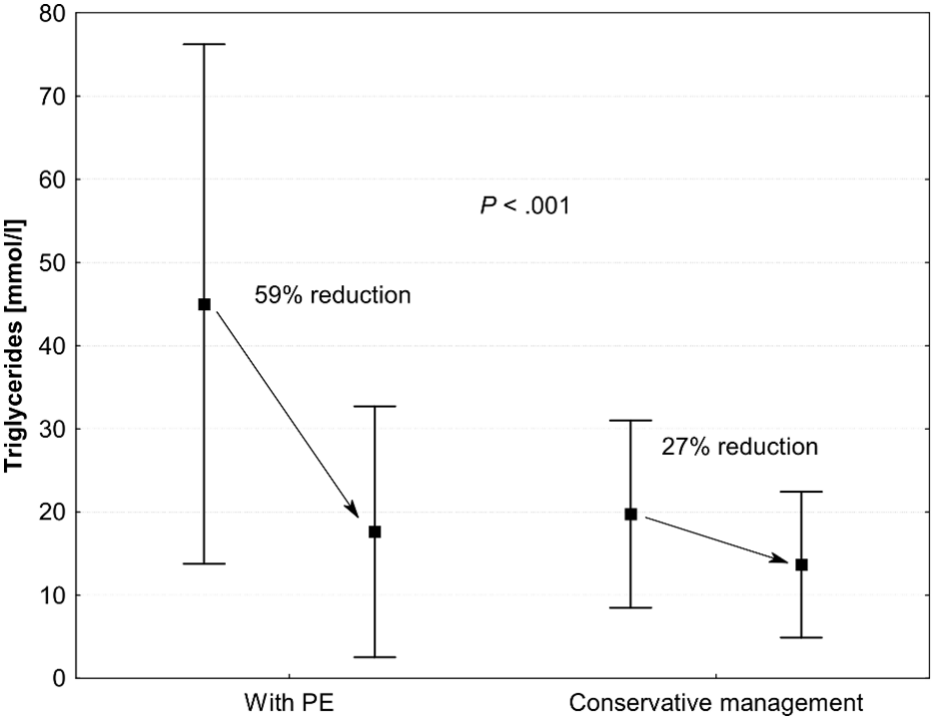
Comparison of the reduction in triglycerides with conservative treatment during 24-hour episodes without plasma exchange (PE) (conservative management, N = 29) and during PE treatments.

The data on lipid measurements during PE procedures are presented in Figure 2. When all PEs were analyzed, triglycerides were lowered from a mean of 44±31 to 18±15 mmol/l (mean reduction of 59%), while total cholesterol was lowered from 17±8 to 10±7 mmol/l (mean reduction of 46%). The reduction in triglycerides was similar in the heparin and citrate procedures after normalization to the volume of plasma exchanged (17±6%/1000 ml vs. 15±22%/1000 ml, p = 0.67). There were 29 episodes of triglycerides above 10 mmol/l which were not treated with PE on the same day. We observed a reduction in triglycerides with conservative treatment from a mean 20±11 mmol/l to 14±9 mmol/l over approx. 24 hours, a mean reduction of 27% (see Figure 3), which was significantly lower than the reduction observed during PE procedures (p<0.001). Similarly, total cholesterol reduced with conservative treatment from a mean of 13±8 mmol/l to 11±7 mmol/l, a mean reduction of 12% (p<0.001 vs. PE).

### Patients’ outcome

The median hospital stay was 16 days (range 2–142), and 68% of patients were hospitalized in the intensive care or step-down unit for some time. Altogether 6 patients died, resulting in a 5% in-hospital mortality rate (see Table 1). Three patients died shortly after admission due to severe shock and multi-organ failure; one had concomitant gastrointestinal bleeding from a Mallory–Weiss tear (estimated by endoscopy to be of minor importance) after PE treatment with heparin anticoagulation. The other three patients died after prolonged hospitalization because of unresolved infections caused either by local complications of pancreatitis or prolonged ventilator dependency; one patient had concomitant pulmonary embolism and lung carcinoma discovered during hospitalization.

### Factors affecting outcome

A comparison of patients’ characteristics in the group of acute pancreatitis episodes in which the patients survived and in the group where patients died is given in Table 2. In addition to APACHE II and calcium, which are known prognostic factors in pancreatitis, sodium was also lower in fatal cases and citrate anticoagulation was rarely used. A comparison of fatal and non-fatal severe cases showed no significant differences. Triglyceride levels at admission were not correlated to APACHE II (r^2^ = 0.002, p = 0.60) or to the length of stay (r^2^ = <0.001, p = 0.77). The median triglyceride level before first PE was 47.4 mmol/l, and the low triglycerides (i.e. ≤47 mmol/l) and high triglycerides groups had only insignificantly different mortality rates (2% vs. 9%, p = 0.10); their length of hospitalization was also comparable (22±18 vs. 19±20, p = 0.36). Patients with severe pancreatitis (APACHE II ≥8) had a significantly higher mortality (24% vs. 1%, p<0.001). When the patients were divided into an early PE group (<36 hours after onset of pain) and a late PE group, there was no difference in mortality (7% vs. 6%, p = 0.79). In 15 out of 21 cases of severe pancreatitis, the delay to first PE could be estimated and the median delay was 30 hours (IQR 26–63). When severe cases were divided into an early PE group (≤30 hours) and a late PE group, there was also no difference in mortality (38% vs. 29%, p = 0.85).

**Table 2:** Comparison of patients’ characteristics at first plasma exchange (PE) in the group of acute pancreatitis episodes in which patients survived (non-fatal episodes) and in the group where patients died (fatal episodes) in all cases and in severe cases only.

Parameter	non-fatalepisodes	Fatalepisodes	P value
**All cases**			
N	105	6	/
Age (years)	47±9	48±7	0.80
Male gender	93/105	4/6	0.35
Triglycerides (mmol/l)	55±34	71±41	0.28
Lipase (µkat/l)	23±34	27±39	0.77
Na (mmol/l)	133±5	129±6	0.03
Ca (mmol/l)	1.90±0.35*	1.37±0.16	<0.01
Creatinine (µmol/l)	89±92	123±40	0.20
C-reactive protein (mg/l)	102±114	139±18	0.58
APACHE II	5±3	10±3	<0.001
Delay from start of painto hospital (hours)	29±42	24±25	0.81
Delay from start of painto first PE (hours)	45±44	43±24	0.91
Citrate anticoagulation	71/104†	1/5†	0.04
Hospital stay (days)	19±14	45±55	0.001
**Severe cases**			
N	16	5	/
Age (years)	48±12	50±5	0.96
Triglycerides (mmol/l)	56±28	75±44	0.25
APACHE II	9±2	10±3	0.29
Delay from start of painto first PE (hours)	41±20	43±26	0.84
Citrate anticoagulation	9/15†	1/4†	0.30
Hospital stay (days)	26±20	52±58	0.13

*N = 38.

† Two episodes of pancreatitis where patients received both heparin and citrate PE were excluded from this analysis; one of the patients died.

Given the significant difference in the type of anticoagulation used during PE in the fatal and non-fatal cases, we compared pancreatitis episodes in regard to the anticoagulation used during PE (see Table 3). Both groups were comparable in all major patients’ characteristics, except serum sodium, which was significantly lower in the citrate group. The group with citrate anticoagulation had a significantly lower mortality (1% vs. 11%, p = 0.04). When only patients with severe pancreatitis were analyzed, the mortality difference remained (1/10 (10%) vs. 3/9 (33%)), but was no longer statistically significant (p = 0.30), probably due to the small number of severe cases. We used a logistic regression model to predict mortality and included significant predictors from univariate analysis (sodium, APACHE II, citrate anticoagulation), except calcium, for which more than half of the values were missing. We excluded two patients who had received both citrate and heparin anticoagulation from this analysis, one of whom died. The model was significant (p = 0.00002) and APACHE II (b = 1.08, p = 0.018) was an independent predictor of a worse outcome, while citrate anticoagulation (b = −6.05, p = 0.049) predicted a better outcome. Since there were only a few deaths and one of the excluded patients died, we additionally used the most conservative approach and included the two patients receiving both modes of anticoagulation in the citrate group; the logistic regression model showed all three variables to be significant predictors, and the p value for citrate even decreased slightly (p = 0.03).

**Table 3:** Comparison of patients’ characteristics and survival in citrate and heparin anticoagulation groups.

Parameter	Heparin group	Citrate group	P value
N*	37	72	/
Age (years)	47±7	47±10	0.85
Male gender	92%	85%	0.37†
Triglycerides (mmol/l)	52±40	56±29	0.59
Sodium (mmol/l)	135±5	132±5	0.02
Calcium (mmol/l)	1.80±0.36‡	1.88±0.38‡	0.52
APACHE II	5±3	5±3	0.77
APACHE II ≥8	24%	14%	0.19†
Delay to first PE (hours)	46±19	45±49	0.93
Stayed in ICU/step-down unit	65%	68%	0.74†
Hospital stay (days)	21±19	20±19	0.80
Hospital stay in survivors (days)	21±17	18±12	0.38
Mortality	4/37 (11%)	1/72 (1%)	0.04†

^*^Two episodes of pancreatitis where patients received both heparin and citrate PE were excluded from this analysis; one of the patients died.

† Fisher exact two-tailed test.

‡ N = 15 for heparin and N = 26 for citrate group.

## Discussion

This is, to our knowledge, the largest observational study on PE treatment in hypertriglyceridemic pancreatitis. The large number of cases included in the study enabled us to observe a seasonal variation in the incidence of hypertriglyceridemic pancreatitis, which probably corresponds to periods of increased intake of fatty foods and alcohol during holidays and outdoor activities, including picnics. Overall, we observed a low mortality rate similar to that of other large cohorts [2], [18], which increased significantly in cases of severe pancreatitis, confirming previous observations.

There are few data available on the recurrence rate after the first attack of hypertriglyceridemic pancreatitis. We have observed a relatively low recurrence rate of 1.6% per year; a similar recurrence rate can be estimated from the study of Athyros [23] (1 recurrence in 17 patients over a mean of 42 months of observation, i.e. approximately 1.7% per year). In a large epidemiologic study [24], the incidence rate of acute pancreatitis in patients with hypertriglyceridemia above 5 mmol/l was also within a similar range (1.09 per 100 patients-years); the incidence rate was halved in patients with triglycerides below 5 mmol/l.

Although hypertriglyceridemia is implicated in the induction and progression of pancreatitis, we did not find triglyceride levels at presentation to correlate with disease severity (i.e. APACHE II), or to significantly influence mortality or length of hospital stay. In accordance with our observations, Balachandra and co-workers also found that slightly elevated triglycerides in acute pancreatitis of various etiologies are not associated with the severity of the disease or its outcome [5], though other authors do report triglycerides to be associated with the severity of pancreatitis [25], [13].

When assessing the value of PE in the treatment of hypertriglyceridemic pancreatitis, one should take into account that triglyceride levels decrease spontaneously with conservative treatment, although the two treatments have not yet been compared in a controlled study. With some, although limited, data on reductions in triglycerides with conservative treatment, we found PE to be twice as effective in reducing triglycerides. It should be noted that PE reductions were measured mainly on the first day or two, while reductions with conservative treatment were observed later in the course of the disease, and the decrease in triglycerides is not necessarily linear. In our cohort, triglycerides were below 10 mmol/l within a median of 2 days of treatment, which is one day sooner than in small case-series treated with heparin and insulin [14], [26]; if triglycerides had been measured immediately after PE and not only the morning after, we would probably have reached this goal at least another half-day earlier. So PE can be expected to lower triglycerides faster than conservative management, although it remains unclear whether this also reduces mortality.

Furthermore, the early institution of PE treatment might be crucial and PE performed too late might not bring benefit any more. Chen published the only comparative study of PE vs. no PE treatment in hypertriglyceridemic pancreatitis, which showed no improvements in mortality after the availability of PE at their institution, although the number of patients actually receiving PE was low [18]. Chen hypothesized that earlier intervention might bring more benefit. All patients in our study received PE and, contrary to expectations, we found no differences in the outcome of patients who received PE early vs. late, either in the whole group or in cases of severe pancreatitis. It should be noted that, overall, our cohort of patients received PE relatively quickly after the onset of pain (median delay of 36 hours, of which one half represents the time needed to reach the emergency department) compared to other studies; e.g. in Chen’s cohort, PE was performed 3 days after the onset of pain [18], and in Yeh’s study 2 days after symptom onset [16]. In spite of the promptness of PE treatment in our patients, mortality was similar to Chen’s cohort [18].

In favor of PE treatment, it should be recognized that there is a very low rate of complications during PE treatment. In our cohort, there were a few cases of hypotension and hypocalcemia (during citrate anticoagulation), and some rare clotting problems due to high blood viscosity. Similarly low incidences of adverse effects during apheresis procedures are reported from the world apheresis registry, with the exception of hypocalcemia in centers where calcium substitution is not routinely used [27], [28].

A recent review states that there is no data to recommend an appropriate replacement solution during PE [8]. All PE procedures in our cohort were done with a 30 g/l albumin solution. More complications can be expected during PE procedures when fresh frozen plasma is used for replacement [27], [29], and since consumption coagulopathy is not a problem (due to the low number of procedures performed in a single patient) and plasma infusion per se was not shown to be beneficial in hypertriglyceridemic pancreatitis [30], we believe there is no need to substitute fresh frozen plasma, and albumin solutions are adequate.

Unexpectedly, we found a significantly lower mortality in the group with citrate anticoagulation during PE as compared to standard heparin. We are aware that this could be a coincidental finding, since this was not a randomized study, but rather a historic cohort comparison, which may be confounded by other differences in clinical practice and supportive care. In accordance with the change in our center’s policy, all PEs in recent years were done using regional citrate anticoagulation as opposed to the previous policy of using citrate anticoagulation only when heparin was contraindicated. Since all six deaths were from the 2003–2008 cohort (published previously [19], approximately at the time of policy change) and no deaths were observed in the later period, this resulted in the above-described mortality difference. Nevertheless, both groups were comparable (except in sodium, which was unfavorably low in the citrate group), and citrate anticoagulation showed an independent positive effect also in the multivariate model. Although heparin itself is used in the treatment of hypertriglyceridemia, it may exacerbate hemorrhage into the pancreatic bed or elsewhere; e.g. in the study by Chen [18] there was a trend towards more upper gastrointestinal bleeding in the group with heparin PE, although it is not clear whether the conservative treatment also may have included heparin infusion. Given the low incidence of complications (i.e. hypocalcemia) during citrate anticoagulation, the efficiency of anticoagulation, and in light of our new observations, citrate might be the preferred anticoagulant during PE in hypertriglyceridemic pancreatitis.

In attempting to explain the positive effects of citrate anticoagulation, one must acknowledge that extracorporeal circuits cause activation of hemostasis and the complement and immune systems. The effects are most pronounced during cardio-pulmonary bypass [31], where a systemic inflammatory response syndrome (SIRS) can develop, but also occur in hemodialysis [32], [33] and apheresis procedures [34], [35], which operate at much lower blood flows resulting in less blood being processed and coming in contact with artificial surfaces. Citrate anticoagulation (as compared to heparin) was shown to increase the biocompatibility of hemodialysis procedures by reducing leukocyte and complement activation [32], [33], [36], [37]. The positive effects are probably mediated by hypocalcemia in the extracorporeal circuit, which reduces many of the calcium-dependant blood-surface interactions. Recently, there was even a report of increased survival with citrate anticoagulation in patients with acute kidney injury requiring continuous hemodialysis [38], although this was not confirmed in another large study [39]. The benefit was most pronounced in sepsis, severe organ failure and post-operatively [38], and could partially be attributed to the increased biocompatibility of the procedure. Although small volumes of blood are processed in PE due to low blood flow and shorter duration, biocompatibility might still be an issue, even more so in a setting of acute pancreatitis, where there is systemic activation of inflammation.

## Conclusions

In acute hypertriglyceridemic pancreatitis, the level of triglycerides at presentation did not correlate with disease severity or influence outcome. One to two plasma exchanges effectively reduced serum triglycerides faster than could be expected with conservative treatment. Plasma exchange treatment is associated with a low rate of complications. Overall mortality was low (5%), increased significantly in severe cases, and was not influenced by the delay in PE therapy. We found that the use of citrate anticoagulation during PE was independently associated with reduced mortality, which should be confirmed in a randomized study.

## Supporting Information

Table S1 **Raw patients’ data.**
(XLS)

Table S2 **Data on all plasma exchange procedures.**
(XLS)
